# Prediction of anemia in real-time using a smartphone camera processing conjunctival images

**DOI:** 10.1371/journal.pone.0302883

**Published:** 2024-05-13

**Authors:** Leon Zhao, Alisa Vidwans, Courtney J. Bearnot, James Rayner, Timmy Lin, Janette Baird, Selim Suner, Gregory D. Jay

**Affiliations:** 1 The Warren Alpert Medical School, Brown University, Providence, Rhode Island, United States of America; 2 Rhode Island Hospital, Providence, Rhode Island, United States of America; Shoklo Malaria Research Unit, THAILAND

## Abstract

Anemia is defined as a low hemoglobin (Hb) concentration and is highly prevalent worldwide. We report on the performance of a smartphone application (app) that records images in RAW format of the palpebral conjunctivae and estimates Hb concentration by relying upon computation of the tissue surface high hue ratio. Images of bilateral conjunctivae were obtained prospectively from a convenience sample of 435 Emergency Department patients using a dedicated smartphone. A previous computer-based and validated derivation data set associating estimated conjunctival Hb (HBc) and the actual laboratory-determined Hb (HBl) was used in deriving Hb estimations using a self-contained mobile app. Accuracy of HBc was 75.4% (95% CI 71.3, 79.4%) for all categories of anemia, and Bland-Altman plot analysis showed a bias of 0.10 and limits of agreement (LOA) of (-4.73, 4.93 g/dL). Analysis of HBc estimation accuracy around different anemia thresholds showed that AUC was maximized at transfusion thresholds of 7 and 9 g/dL which showed AUC values of 0.92 and 0.90 respectively. We found that the app is sufficiently accurate for detecting severe anemia and shows promise as a population-sourced screening platform or as a non-invasive point-of-care anemia classifier.

## Introduction

Anemia, defined as a low blood hemoglobin (Hb) concentration, has a global prevalence estimated at 22.8%, or 50.3 million years lived with disability in 2019 [1]. Young children, menstruating women, and pregnant and postpartum women are particularly affected by iron deficiency anemia [2]. The World Health Organization (WHO) estimates that 40% of children under the age of five in 35 countries have anemia and has classified anemia as a severe public health problem [3]. Anemia can result in a range of clinical complications, such as immune system dysfunction, impaired thermoregulation and neurocognitive function, and slow cognitive and motor development. Understanding the socioeconomic determinants and developing new tools for early diagnosis should remain a priority.

The standard test for diagnosing anemia is the Complete Blood Count (CBC). While a CBC measures the severity of anemia, it requires venipuncture, trained phlebotomists, laboratory technicians, the use of chemical reagents, and dedicated lab equipment [4]. In resource-rich hospitals, it takes 1–4 hours to obtain results. Therefore, the collection of CBC is often limited to geographical regions with adequate healthcare infrastructure. However, studies have shown that anemia is disproportionally prevalent in rural areas that lack these resources and visualization of conjunctival pallor is relied upon to qualitatively support anemia but is unreliable [5, 6]. As a result, there is an unmet need for accessible, non-invasive, point-of-care (POC) tools that can screen for anemia rapidly. Non-invasive measurement of Hb [4, 7–10] has been made possible through smartphone imaging of the palpebral conjunctiva [4, 7–10]. The light reflected off the palpebral conjunctiva membrane can be evaluated by digital photographs, and studies have shown that images in RAW [4] and RGB format [7–10] obtained from a smartphone camera can predict Hb concentration, albeit imprecisely. Apps that process nail-bed images are similarly imprecise upon real-world testing [11, 12]. Lack of precision using a smartphone app is outweighed by the sheer accessibility of smartphones to the general population. The ability to assess more patients through the ubiquity of cell phones presents a near-term opportunity for universal screening for anemia through telehealth apps.

Studies of POC devices for non-invasive Hb prediction appeared promising based on initial foundational data sets but were not validated in many instances [7, 13–15] which are summarized in S1 Table. Measuring Hb concentration post-hoc from palpebral conjunctiva images has a reported accuracy of 82.9% in an adult population [4]. Other studies using cell phone enabled apps reporting Hb in real time [8, 9, 16] report similar results (S1 Table). Given the previous limitations in accuracy, our study aimed to evaluate a self-contained app and user interface as a tool for anemia severity classification in real-time using RAW images processed in a 32 bit enabled classifier. The novelty and innovation of the present work is to show that a self-contained POC device can estimate Hb concentration around critical and actionable transfusion thresholds by imaging an area that physicians are already familiar with- the conjunctiva. Determining the urgent need for blood transfusion is a suitable gateway for early adoption of this computerized app by emergency and intensive care medical personnel. In these situations, the hypothetical risks of empirical measurement may be counterweighted by the risk of failing to appreciate patients’ severe anemia. The app is also user friendly in so far that self-measurement is possible.

## Materials and methods

### Study design

This was a prospective observational validation study of a convenience sample of adult patients presenting to the Emergency Department (ED) between June 18, 2022 and February 10, 2023. Patients over the age of 18 years presenting with any chief complaint and having 1) a CBC obtained as part of their medical care, 2) the ability to provide informed consent, and 3) the ability to expose the palpebral conjunctiva of both eyes, were approached for participation. Patients with injury, jaundice, inflammation, or infection of either eye were excluded. This study followed the Standards for Reporting of Diagnostic Accuracy guidelines (STARD) 2015 [17], which provides a checklist of essential items for reporting diagnostic accuracy studies.

### Study population

**Fig 1:**
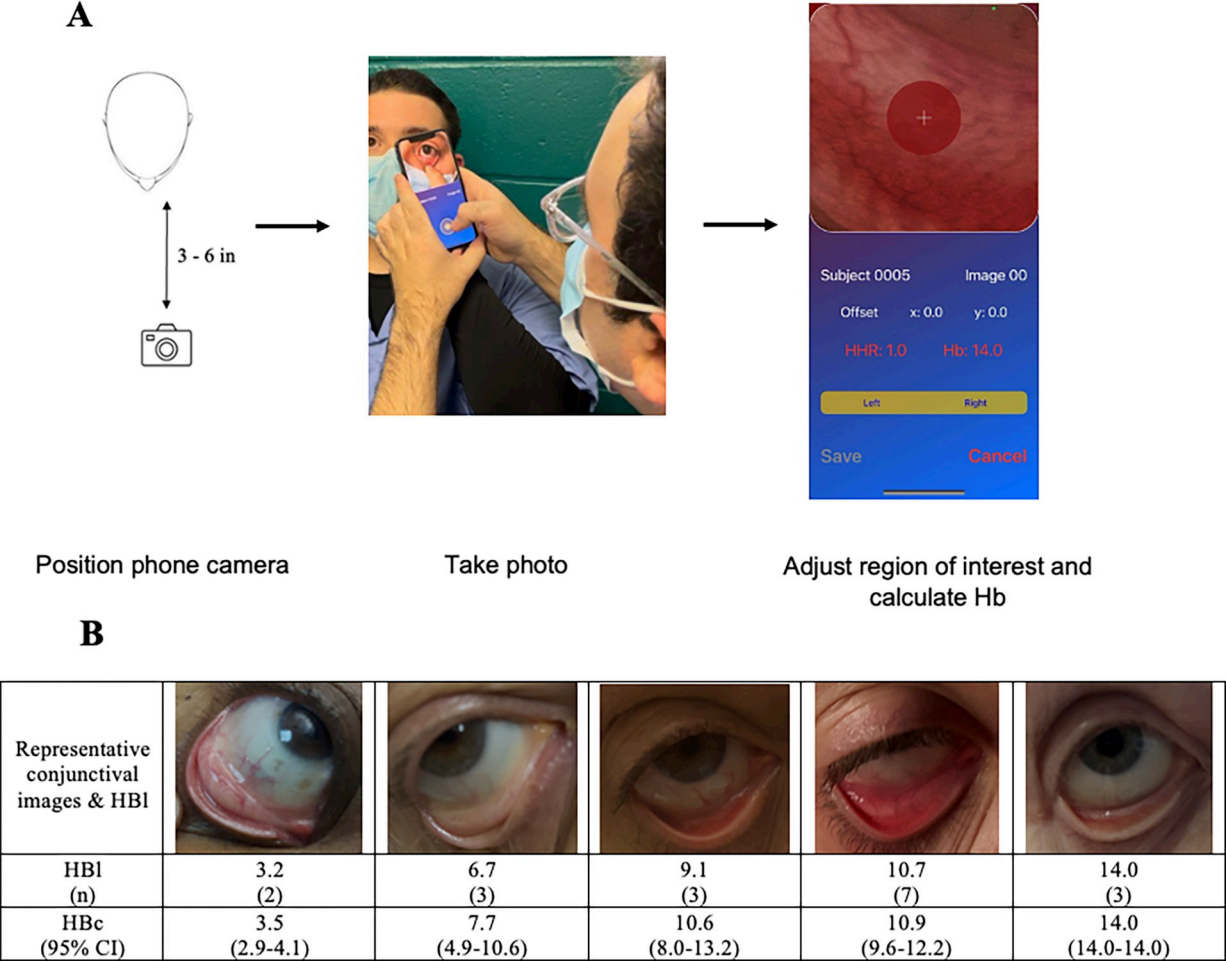
Smartphone app interface and camera positioning. Demonstration of use of an eMoglobin app enabled smart phone on a volunteer member of the research team, A) 3–6 inches away depending upon ambient lighting and image clarity (B) representative captured images and HBc estimated Hb values.

Lifespan Institutional Review Board approved this study (Ref # 209416). All patients who participated provided written informed consent. The individual pictured in Fig 1 has provided written informed consent (as outlined in PLOS consent form) to publish their image alongside the manuscript. Participating patients were identified based on a Best Practice Alert (BPA) in the EPIC electronic medical record that notified research assistants (RA) when a patient had an abnormal Hb level as determined by their CBC. Abnormal was defined as <13.5 g/dL or >16.0 g/dL for men and <12.0 g/dL or >15.0 g/dL for women. Research assistants consented and enrolled participants, and then acquired conjunctival images within 4 hours of venipuncture for CBC determination.

Demographic information was collected along with hospital laboratory-reported test results including blood oxygen saturation, HBl (laboratory-determined Hb), and time of collection. The specific causes of anemia were not a subject of study and thus were not included in the database.

### Anemia classification binning

Five anemia classifications were identified: mild anemia (≥ 10 and < 12 g/dL) for women, mild anemia (≥ 10 and < 13.5 g/dL) for men [18], moderate (≥ 7 and < 10 g/dL) [19], severe (≥ 5 and < 7 g/dL) [16] and extreme (< 5 g/dL) for men and women [20, 21]. The classification bin for mild anemia also included the participants who were normemic (≥ 12 g/dL) for women and (≥ 13.5 g/dL) for men [18].

### Image capture and processing

Participants were asked to remain still and retract their lower eyelids to expose the conjunctiva with the assistance of research assistants (RA) if necessary. Images were obtained in the eMoglobin app on an iPhone X with iOS version 15.6.1 (Apple Inc, Cupertino, CA) under ambient indoor light using the back camera held approximately three to six inches from the target conjunctiva (Fig 1). Tapping the screen to focus on the conjunctiva activated the auto-focus and brightness adjustment of the smartphone camera. A minimum of four images were obtained from each patient: two images of the left and right eye. Images were recorded in RAW format without flash and images were encoded at a 32-bit level of resolution (S1 Fig) [4]. After each image was taken, the app displayed the image for the research assistant to visually inspect and select the region of interest (ROI). The research staff were instructed to adjust the ROI so that the displayed image was centered on the patient’s palpebral conjunctiva. Selected ROI images were stored and processed as described previously [4] and analyzed in the eMoglobin app in real time. High hue ratio was calculated from each image and correlated to the HBl from the previously validated correlation. Conjunctival Hb concentration estimates (HBc) were available instantly [4].

### Statistical methods

Statistical analysis was conducted using SAS (version 9.4, Cary, NC). Descriptive statistics were utilized to characterize the study population by age, race, and skin color [4] and describe the distribution of HBl values. Polynomial regression was performed to assess the association between HBl and HBc values, including a quadratic term for HBc. A regression analysis was also performed to assess the relationship between high hue ratio and HBl. Additionally, a mixed effects regression analysis was conducted with RA as the random effects to assess the variance in the association between HBc and HBl accounted for by RA operator performance (n = 16). Due to variability in the numbers of patients assessed by the RA operators, we categorized this into a 2-level of assessed volume-based categories for RA’s (< 20, ≥20 patients). The intraclass correlation coefficient (ICC) was calculated to assess the operator effect.

### Clinical usefulness of HBc

HBl was categorized as anemic (<12 g/dL for women and < 13.5 g/dL for men) or not anemic as defined by the American Society of Hematology (ASH) [22]. The association between HBc and HBl across different levels of anemia described above was evaluated using 2x2 tables to calculate accuracy, sensitivity, specificity, false positive and false negative rates. Additionally, discrimination was evaluated by the Area Under Curve (AUC) of the receiver operating characteristic (ROC) curves using logistic regression.

### Bland-Altman plots

Bland-Altman plots assessed the agreement between HBc and HBl measures. The Bland-Altman plot depicts the difference between HBc and HBl plotted against the average of HBc and HBl measures.

## Results

During the enrollment period between June 18, 2022, and February 10, 2023, images from 435 unique participating patients were obtained. Data from nine subjects were excluded because each had only one image recorded, leaving 426 subjects for the final analysis. The average age was 53 years (range 19–94) and 55.2% were women. The study population was roughly representative of the racial and ethnic distribution of Rhode Island in the 2020 census [23] - 72.8% White and 12.7% Hispanic or Latinx (S2 Table). The predominant skin color was light (75.6%), 424 patients (99.6%) did not have jaundice, and 413 (97%) had normal oxygen saturation levels (mean oxygen saturation: 97.46%; 95% CI 97.44, 97.47%). Mean HBl was 12.6 g/dL (range 3.2–18.8 g/dL, 95% CI 6.8, 18.3 g/dL) and the distribution of HBl is illustrated in S2 Fig. The average of the four app-derived Hb values was calculated to produce a HBc estimate.

**Fig 2:**
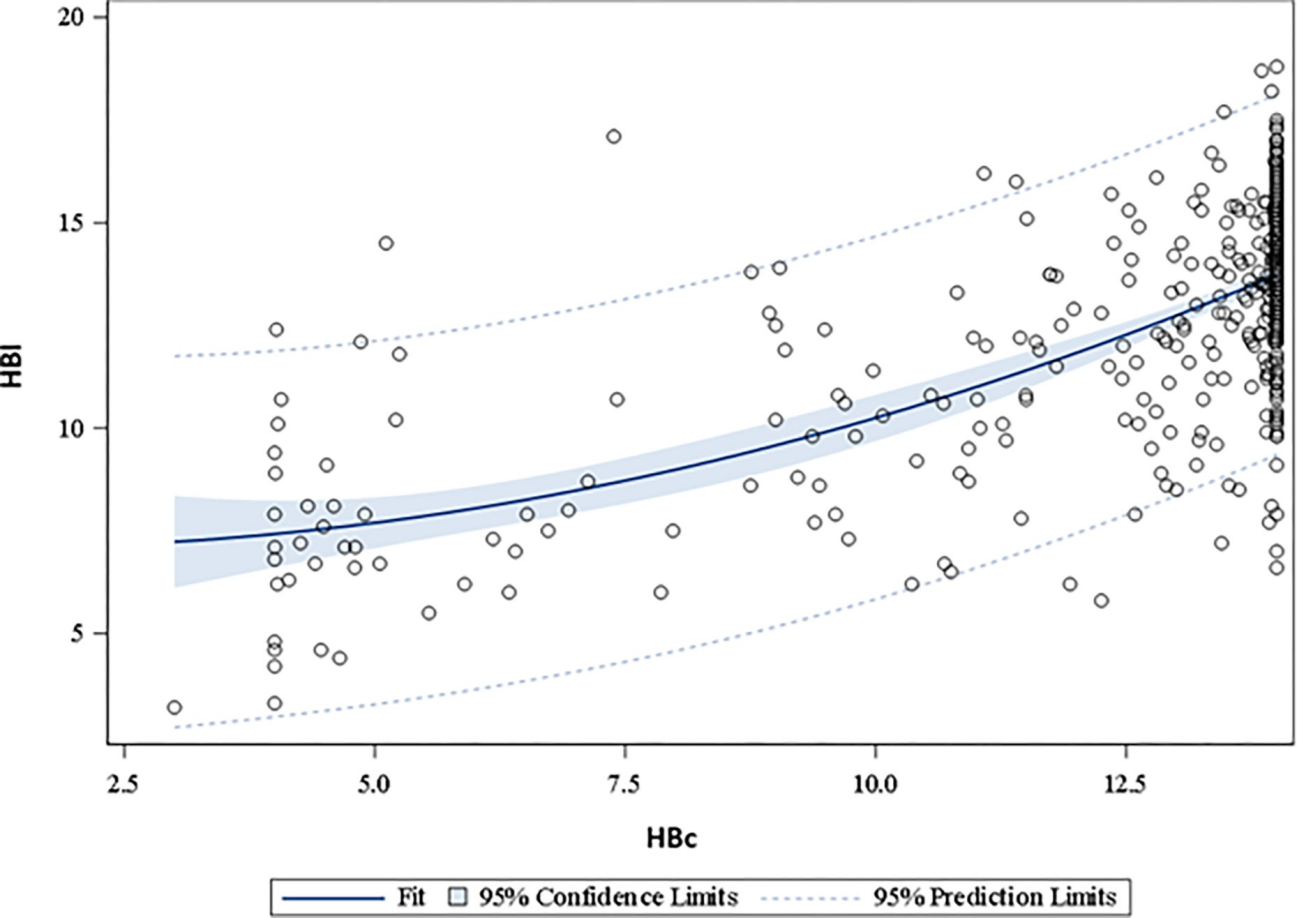
Correlation between HBc and HBl. Correlation between conjunctiva-estimated Hb (HBc) (average of all four replicates) vs actual measured Hb (HBl) in pre-defined operating range (2–14 g/dl). There was a moderate degree of association (p < .001) between HBc and HBl, with correlation coefficients of 0.65 and 0.66 for HBc and the quadratic of HBc respectively. These correlation coefficients do not represent the actual association between HBc and HBl as HBc prediction values were limited to 14 g/dL in the app display, contributing to clustering at HBc at 14 g/dL due to the processing and display limitation of the app which does not execute calculations and prediction is excess of 14 g/dL.

HBc and HBl were plotted and a visual inspection of the best line of fit and residuals (Fig 2), indicated that a quadratic fit best identified the relationship between HBc and HBl. The intercept was = 0 (95% CI -0.072, 0.072), and the quadratic of HBc was 0.66 (95% CI 0.59, 0.73). A slope = 1.0 indicated perfect fit. This suggests that HBc underestimates HBl.

The majority (81.7%) of participants had mild anemia by serology (Table 1A). There were comparatively fewer subjects with severe (3.8%) and extreme (1.6%) anemia respectively. The app was most accurate in the extreme anemia category, with agreement between HBc and HBl being 100% (Table 1B). The app colocalized HBl and HBc estimates in 25.3% of the mildly anemic patients. The HBc estimates incorrectly categorized normemic subjects of both sexes as mildly anemic in 60 of the 95 mildly anemic patients (63.2%). Predictions in the severe (≥ 5 and < 7 g/dL) and moderate (≥ 7 and < 10 g/dL) categories had greater variability, with agreement of 18.2% and 25.0%, respectively (Table 1B).

**Table 1:** Sub-populations of anemia threshold binning. Population frequency of laboratory measured Hb (HBl) values in anemia classifications. B) Agreement of HBc and HBl in participants across anemia classifications.

**A**							
Anemia classification (g/dL)				N (%)			
Normemic (Women: ≥12; Men: ≥13.5)				253 (59.4%)			
Mild Anemia (Women: ≥10 and <12; Men: ≥10 and <13.5)				95 (22.3%)			
Moderate Anemia (≥7 and <10)				55 (12.9%)			
Severe Anemia (≥5 and <7)				16 (3.8%)			
Extreme Anemia (<5)				7 (1.6%)			
**B**							
		HBI (g/dL)					
		Normemic (≥12 for Women and ≥13.5 for Men) (n = 253)	Mild Anemia (≥10 and <12 for Women) and (≥10 and <13.5 for Men) (n = 95)		Moderate Anemia (≥7 and <10) (n = 55)	Severe Anemia (≥5 and <7) (n = 16)	Extreme Anemia (<5) (n = 7)
	Normemic (Women: ≥12; Men: ≥13.5) (n = 306)	227 (89.7%)	60 (63.2%)		18 (32.7%)	1 (6.25%)	0 (0%)
	Mild Anemia (Women: ≥10 and <12; Men: ≥10 and <13.5)) (n = 57)	18 (7.1%)	24 (25.3%)		10 (18.2%)	5 (31.25%)	0 (0%)
HBc (g/dL)	Moderate Anemia (≥7 and <10) (n = 23)	5 (2.0%)	7 (7.4%)		10 (18.2%)	1 (6.25%)	0 (0%)
	Severe Anemia (≥5 and <7) (n = 12)	1 (0.4%)	2 (2.1%)		5 (9.1%)	4 (25%)	0 (0%)
	Extreme Anemia (<5) (n = 28)	2 (0.8%)	2 (2.1%)		12 (21.8%)	5 (31.25%)	7 (100%)

HBl is laboratory-measured Hb and HBc is conjunctival-estimated Hb

Accuracy of HBc was 75.4% (95% CI 71.3, 79.4) for all categories of anemia, and was significantly lower for women (AUC = 0.74) compared to men (AUC = 0.79). Sensitivity and specificity of HBc for predicting anemia in men and women combined was 54.3 (95% CI 46.9, 61.8) and 89.7 (95% CI 86.0, 93.5), respectively. Accuracy, sensitivity, and specificity of HBc for predicting anemia at a high transfusion threshold (<9 g/dL) were greater with values of 91.1 (95% CI 88.4, 93.8), 58.3 (95% CI 45.9, 70.8), and 96.5 (95% CI 94.6, 98.3), respectively. Accuracy, sensitivity, and specificity of HBc for predicting anemia at a low transfusion threshold (<7 g/dL) were greater with values of 92.7 (95% CI 90.3, 95.2), 69.6 (95% CI 50.8, 88.4), and 94.0 (95% CI 91.7, 96.4), respectively (S3 Table).

**Fig 3:**
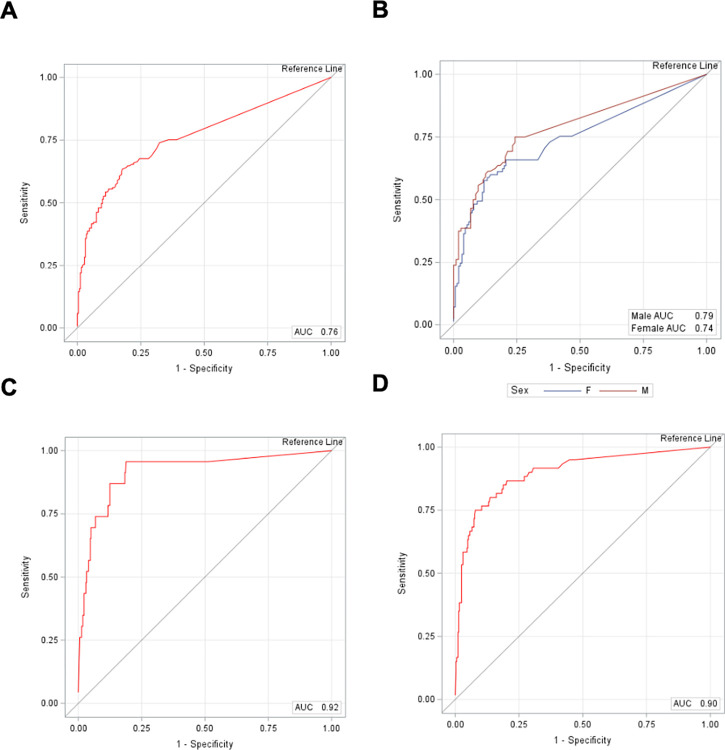
Receiver operator characteristics of HBc using HBl across different anemia guidelines. Receiver operator characteristic curves for A) overall in both sexes using 12 g/dL as the anemia threshold, B) anemia definitions differentiated by <12 and <13.5 g/dL for women and men respectively, C) restrictive transfusion threshold of 7 g/dL defined by the American Association of Blood & Biotherapies (AABB), and D) liberal threshold of 9 g/dL defined by the AABB. The x-axis depicts 1-specificity and the y-axis shows the sensitivity. The red line represents the ROC and the black line is the no-discrimination line.

ROC curves were generated to analyze the effect of increasing transfusion threshold on the sensitivity and specificity of HBc (Fig 3). AUC overall for sex-specific cut-off was 0.76 (95% CI 0.72, 0.81) and the AUC was not significantly different between women and men cut-off values (women = 0.74 (95% CI 0.68,0.81); men = 0.79 (95% CI 0.73,0.85)). AUC for the restrictive transfusion threshold was 0.92 (95% CI 0.85, 0.98), while AUC for the liberal transfusion threshold was 0.90 (95% CI 0.85, 0.94). The present eMoglobin algorithm had greater diagnostic accuracy for Hb values below the restrictive and liberal transfusion thresholds compared to values below the threshold values for anemia for men and women.

**Fig 4:**
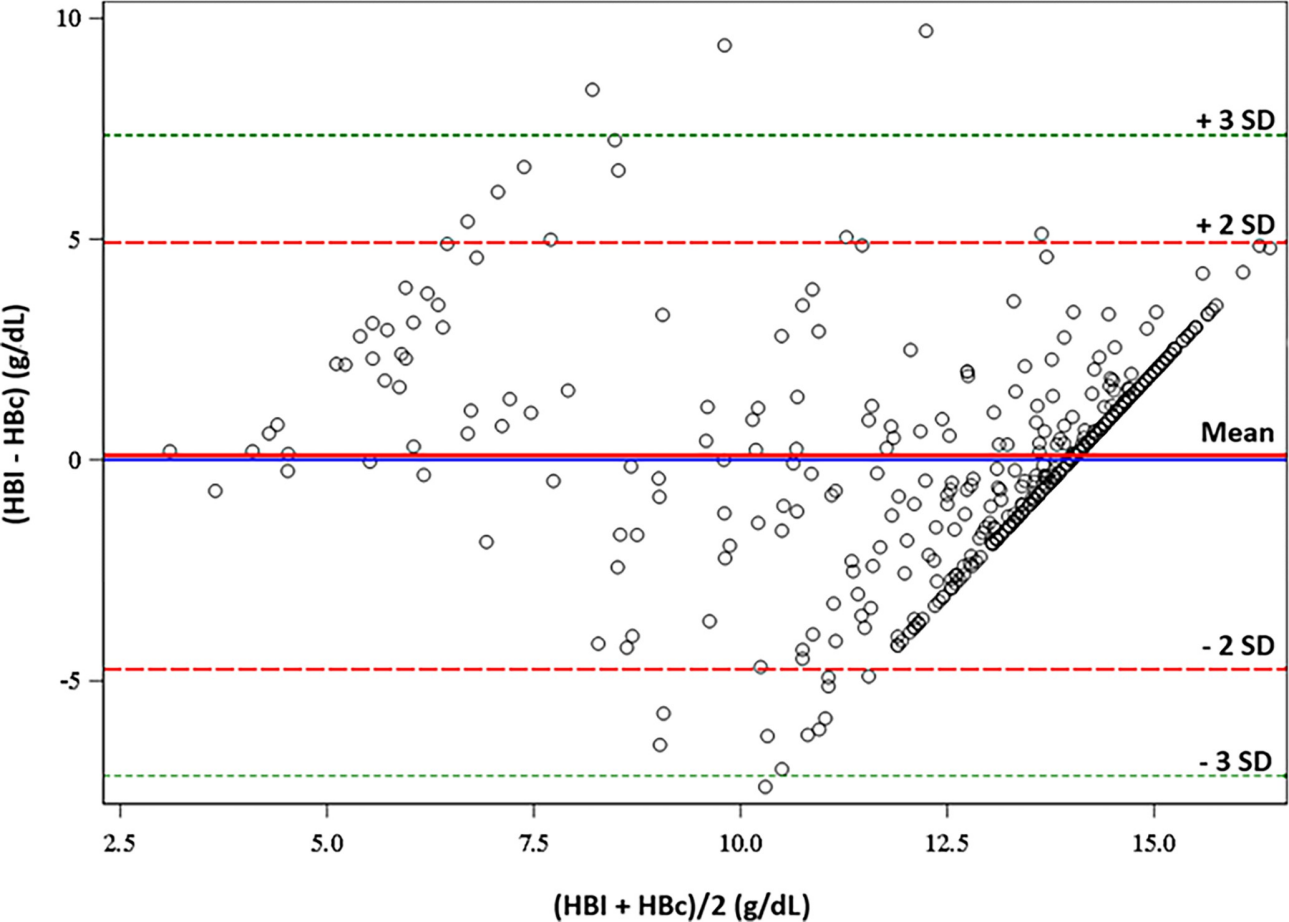
Bland-Altman plot for HBc compared to HBl. Bland-Altman plot for HBc (average of the four replicated estimates) compared to HBl (laboratory-determined) for all 426 participants. The average Hb concentration (HBl+HBc/2) in g/dL on the x-axis is plotted against Hb concentration error (HBl–HBc) in g/dL on the y-axis. The red shaded area represents limits of agreement. The solid red line represents the mean difference between HBl and HBc, +/- 2 standard deviations of the mean of the differences (dashed red line), +/- 3 standard deviations of the mean of the differences (green dotted line), and the solid blue line represents 0 error.

The Bland-Altman plot exhibits clustering along a diagonal line in the higher range of average HBl (>10 g/dL) due to the algorithm which set the maximum HBc to 14 g/dL in the app. Bland-Altman plot analysis shows a bias of 0.10 and limits of agreement (LOA) of (-4.73, 4.93 g/dL). Error was found to trend with increasing average Hb values (slope = 0.68 (0.60, 0.75), p < .001) (Fig 4).

Intraclass correlation calculation between operators showed that using a 2-level category (< 20 patients, ≥ 20), 3.4% of the variance in HBc predicting HBl was accounted for by the RA performance. This indicates that the predictive accuracy of HBc from the eMoglobin algorithm was minimally impacted by interoperator variability (S4 Table).

## Discussion

This is the first study to evaluate the clinical utility of a smartphone-based app employing a novel 32 bit image processing method developed to classify anemia severity using digital images of the conjunctiva processed locally in real time. Anemia is a pressing health concern worldwide and disproportionately affects people living in under-resourced areas [5]. Early screening and detection of anemia is crucial for preventing associated sequelae such as delayed cognitive and motor development in children, impaired immune response, and increased maternal mortality [24]. As smartphones become ubiquitous, a non-invasive smartphone-based tool for anemia screening could serve as a pre-clinical and clinical tool that would allow for timely and inexpensive anemia screening for patients without access to blood testing.

To validate this smartphone app we used a data set that was collected separately from a previously reported training and validation set [4]. A separate validation study is an important requirement in developing these devices to avoid data overfitting where noise in the derivation data set negatively affects the accuracy of the device. Validation studies using a new cohort of data have been reported in only four studies developing smartphone-based apps for Hb estimation (S1 Table). In the present study, participant data was collected in an ED clinical setting and test results were compared to blood-drawn standard measurements. Our app relied on the smartphone camera for *in vivo* image collection and did not require external smartphone attachments [10, 25]. This enables anyone with a smartphone and internet connection to use the app. Our algorithm utilizes pixel extraction instead of analysis of image RGB values. In previous methods that used RGB value analysis, which a majority of other Hb prediction apps use [7–10, 13–15, 26–28], each pixel has a red, green, and blue value that contributes to the color of the image and is limited to one of 256 values [28]. Using techniques described by Sumner [29], a RAW image processing algorithm that maximized the color resolution of images was developed, wherein each color level was encoded with 32 bits giving 4.3 trillion values for each channel and 8x1028 colors allowing for highly accurate color analysis. These data were further processed into a high hue ratio [14] which appeared to be more closely approximating Hb values (S3 Fig). Consistent with our previous study using the same algorithm [4], the use of an artificial light source did not appear to change the quality of images and affect prediction accuracy.

Due to previously published limitations in the predictive accuracy of smartphone-imaged conjunctiva for Hb [4, 7–10], we chose to analyze the predicted Hb values in a role as a screening device for anemia classification. Discretization of a continuous clinical variable into intervals in theory should partition and maintain original patterns and recapitulate how the data would be interpreted by the end user, and may also minimize spurious data [30]. For patients with severe anemia, the app had higher accuracy, sensitivity, and specificity compared to patients with moderate anemia. These findings suggest that the eMoglobin app is more suitable for detecting severe anemia rather than mild or moderate anemia. This is useful clinically as detection of severe anemia is more time-sensitive than identifying mild anemia.

Several apps in development appear promising based on imaging of perfused tissue surfaces but underperformed compared to co-oximetry and invasive methods [31, 32]. Fingernail bed imaging [16] was based on an imaging algorithm used for drawn blood [11]. However when applied non-invasively to fingernail surfaces in Bihar, India to predict Hb values in a population of school-aged children the app proved to be highly inaccurate without algorithm training [12]. The benefit of basing Hb prediction on images of the conjunctiva [26] compared to other vascularized surfaces such as fingernails is that vascular beds are not affected by factors including poor perfusion from vasoconstriction and shock, fungal infections, discoloration from trauma, fingernail polish and staining, or artificial fingernails [16].

The rise of point-of-care digital health technologies has been catalyzed by the SARS-CoV-2 pandemic and the mobile health market size is expected to reach $189 billion by 2025 [33]. However many mobile health smartphone apps are unregulated and pushed to market before rigorous validation [34, 35].

Numerous thresholds in classifying anemia exist in the literature [18–20, 36–38]. Reexamination of a normal Hb level associated with the overall clinical well-being of patients in low and middle-income countries (LMIC) results in a level lower than the WHO 12.0 or 13.0 g/dL criteria for women and men respectively [37]. The National Cancer Institute cites mild anemia as 10 g/dL to the lower limit of normal [18]. This range-based definition of mild anemia is supported in both LMIC [21] and high-income countries [38]. Thresholds for severe anemia coincide with thresholds for transfusion which are stratified as either restrictive (7–8 g/dL) or liberal transfusion (9–10 g/dL) thresholds in moderate to severe anemia often depending upon accompanying symptoms and co-morbidities [36]. In Kigali, Rwanda for example 5 g/dL is the recommended threshold below which blood transfusion is recommended [20]. In the UK and US this therapeutic recommendation is anchored at 7 g/dL [36]. Thus, the differentiated bins in this investigation were established *a priori* in an approach to address the myriad of anemia definition thresholds and associated blood transfusion guidelines from a global perspective.

The app’s LOA is comparable to that of the Radical-7™ pulse co-oximetry device (Masimo, Irvine, CA) when first reported, and is a FDA-approved POC Hb measuring device (Bias -1.09, LOA -4.20 to 2.02) [39]. The Masimo Pronto pulse co-oximeter has a sensitivity and specificity of 86% and 81% respectively in measuring anemia at a threshold of 13.5 g/dL [40].

The present eMoglobin algorithm or other modes [31] of perfused tissue reflectance spectrophotometry [4] may be useful in the screening for severe anemia in populations or patients at risk, respectively in-home use/self-care applications. If it were accepted as an inexpensive screen, we propose that this metric would be abbreviated as SrHb to quickly communicate ‘reflective spectrophotometry’ as the method of operation for Hb estimation and its inherent limitations. This abbreviation conveys the similar (but unrelated) abbreviation of SpHb which refers to continuous Hb monitoring provided by pulse co-oximetry.

The results of this study should be interpreted in the context of certain limitations. First, RAs had to confirm that a patient’s CBC was taken within the four-hour study window. Thus, RAs were not blinded to the HBl which could have introduced selection bias when considering the ROI. Secondly, despite tailoring the enrollment strategy to target patients with moderate to extreme anemic thresholds, only 78 (18.3%) of participants met these thresholds. The study likely would have benefitted from enrolling more severely anemic patients to increase the statistical strength of the specificity calculations. Thirdly, lighting was not rigorously controlled and some variation in Hb estimation is possible. Future work would include efforts to understand intra-subject variation such as studying participants undergoing blood transfusion with repeated measures.

## Conclusion

The findings from this study demonstrate that a smartphone app can serve as a useful tool for non-invasive severe anemia classification with acceptable accuracy. Given the high prevalence of anemia and its associated morbidity and mortality, the development of an accurate and accessible anemia screening tool is essential to reduce the risk of health complications and alleviate the social and economic burdens associated with untreated anemia. This app may have substantial clinical utility in a myriad of settings in which noninvasive anemia screening would be beneficial.

## Supporting information

S1 Fig Technical description of eMoglobin algorithm. (DOCX)

S2 Fig Distribution of laboratory-determined Hb (HBl) values. Subjects’ HBl levels ranged between 3.2 and 18.8 g/dL (mean 12.6 g/dL) across N = 426 participants.
(TIF)

S3 Fig Average high hue ratio vs HBl. The high hue ratio (HHR) primary parameter extracted from the images was the most predictive of HBI. HHR values were correlated with HBc range of 2 to 14 g/dL The mean HBl intercept was 5.8 (4.9, 6.7) (95% CI; p<0.001). The mean HHR was 7.6 (6.6, 8.5) (95% CI; p<0.001). Value of mean std error was 5.47 and R-squared was 0.372.
(DOCX)

S1 Table Referenced studies of methods to estimate Hb concentration non-invasively by imaging perfused tissue surfaces. (DOCX)

S2 Table Patient demographics. (DOCX)

S3 Table Clinical usefulness of conjunctiva-estimated Hb (HBc). Accuracy, sensitivity, specificity, false positive rate, and false negative rate for anemia as defined by the American Society of Hematology (ASH) and for transfusion thresholds as defined by the Association for the Advancement of Blood & Biotherapies (AABB) are shown as predicted values [95% CI].
(DOCX)

S4 Table Intraclass correlation calculation for RAs. Intraclass correlation calculation using a mixed effect regression analysis predicting laboratory-determined Hb (HBl) measurement using the eMoglobin data.
(DOCX)
