# Supplementary material for: Prediction of anemia in real-time using a smartphone camera processing conjunctival images
Source: PLoS One. 2024 May 13;19(5):e0302883. doi: 10.1371/journal.pone.0302883 (PMC11090304; doi:10.1371/journal.pone.0302883)
Supplement: S1 Fig — (DOCX) [file pone.0302883.s001.docx]

**Technical Description of eMoglobin Algorithm**

The block diagram below (Fig 1) summarizes the steps in the process of an iPhone app to noninvasively measure hemoglobin (Hb).

**
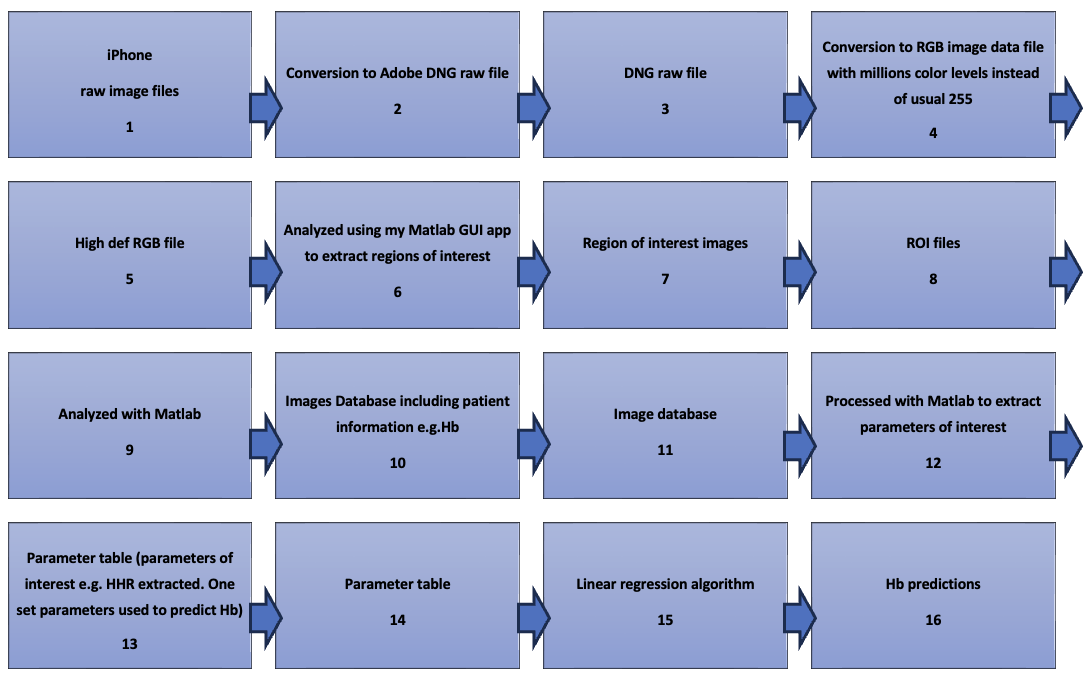
**

Fig 1. Block diagram of algorithm programming responsible for image processing and Hb prediction

**Initial Image Processing (1-5)**

RAW image files generated by the iPhone camera were used for analysis. These files contain data generated directly from the camera sensors with minimal processing. These are used preferentially over the standard RGB format which has been processed.

When used to estimate hemoglobin concentration we endeavored to render as much color accuracy as possible so an algorithm was developed that converts an N x M pixel RAW image to 3 N x M matrices where each matrix represents the red, green, and blue components of an RGB color image. In lieu of 8 bits there are 32 bits per component or approximately 4.3 billion levels per component and a resulting 10^29^ possible colors. This system enhances sensitivity to color variations and leads to increased accuracy of Hb predictions. The total number of possible colors is therefore 256x256x256 or roughly 16.8 million colors, which is more than the human eye can perceive. By contrast, typical RGB images have 8 bits for each component meaning each color component can range between 0 and 255.

**Region of Interest Extraction (7-10)**

Currently the system extracts the correct region for processing given a user-selected seed point, that is a user-selected point defined in the region of interest (ROI). Selecting one point is not a great burden and in our app crosshairs on screen will help ensure the user is lined up with conjunctivae correctly. The crosshair intersection will then be used as a seed point so region selection is automated. The algorithm uses the seed point and branches out to similar neighbors in a pseudo-crystallization paradigm to select the pixels of interest (Figure 2).


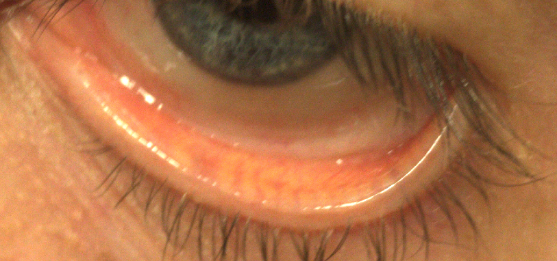

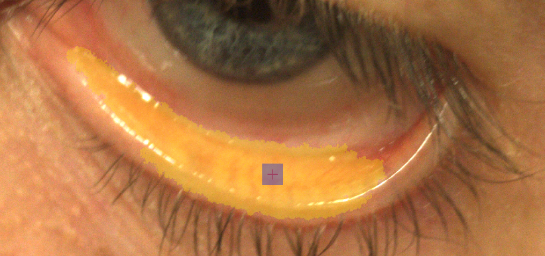


Figure 2. Selection of a seed point (red cross) on conjunctiva leads software to extract ideal region for analysis (shown in yellow). This automated system also autocorrects for lighting conditions by performing an automated white balance against the sclera.

**Image Database (8-11)**

Production of an efficient hemoglobin prediction model requires repeated model development and testing. To speed up this process, an algorithm in Xcode takes raw image files and then stores the information from the seed point extraction steps in a database. Each database entry contains a link to the raw file, extensive image metadata, and an image of the region of interest in high-color resolution format. There are currently approximately 2000 images from 150 patients each with hemoglobin measured by blood draw in the development of a validated “look-up table”.

**Feature Extraction (11-14)**

Each region of interest image in the database varies in size but typically has approximately 50,000 pixels. The color of each pixel is defined by three numbers so that each image is stored as approximately 150,000 numbers in three 2-dimensional matrices. In addition, there is more information from the camera itself as digital images typically store a variety of metadata. These metadata give us access to information such as white balance.

Features are extracted from high-definition image data to produce a set of parameters. These parameters are then used to construct a mathematical model to predict hemoglobin. For example, a parameter could be the average value of the red component of all pixels in the image.

There are 22 parameters for each image that encapsulate the information stored in the image itself as well as information from camera metadata. These 22 parameters are generated for each image in our database creating a Parameter Table. Each row in the table contains the 22 parameters for a specific image plus the measured hemoglobin from blood draw used as the gold standard. Initially, parameters were selected based on features proven to be useful in previous research, for example, Chen et al. (Cited Ref #14 in References).

Using our image database we now can generate and test new parameters easily and refine our parameter set, by testing the information content of each parameter, to improve accuracy and efficiency. Ideal parameters would vary greatly with hemoglobin levels while not being redundant. Redundancy occurs due to correlation between parameters. For example, average red value of an image is a useful parameter but average green and blue levels often mirror red. Having all three as parameters therefore adds less information than one would expect and we can improve efficiency by dropping such redundant parameters.

**Construction of Prediction Model (14-16)**

Using our chosen parameters created from features extracted from each digital image, the Parameter Table was updated. Each row has a set of parameters for a given image as well as the actual measured hemoglobin for that subject.

The Parameter Table was used to construct mathematical models to predict hemoglobin given a set of parameters. We have implemented a number of linear regression models including stepwise methods to reduce redundancy of parameters, and robust models that better tolerate noise in the data.

K-fold testing is used to create predictions for each image allowing error to be calculated when compared to measured hemoglobin. We use 10 folds in our model testing. This means 10 percent of the dataset is randomly selected as the test set and the model was trained using the remaining 90%. The model is then tested with the 10% test set. This process is repeated to identify the best models and identify image properties which improve or worsen hemoglobin prediction. This approach has resulted in the following equation that is predictive of Hb concentration: Hb = 4 + 10*HHR where HHR is high hue ratio. HHR = nH / N where nH = number of pixels with hue over threshold level and N = total number of pixels.
