# Supplementary figures and images for: Prediction of anemia in real-time using a smartphone camera processing conjunctival images
Source: PLoS One. 2024 May 13;19(5):e0302883. doi: 10.1371/journal.pone.0302883 (PMC11090304; doi:10.1371/journal.pone.0302883)

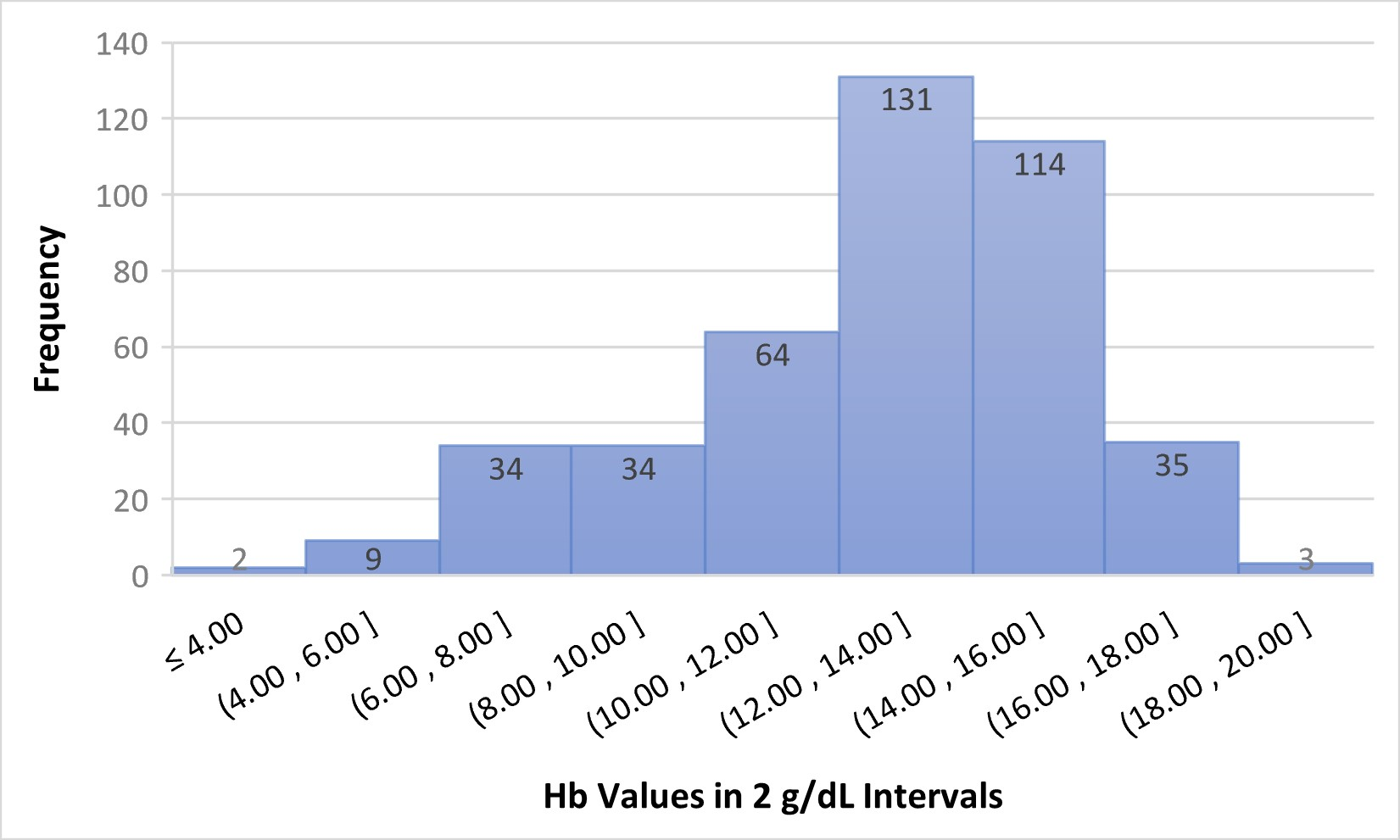

Supplement: S2 Fig — Subjects’ HBl levels ranged between 3.2 and 18.8 g/dL (mean 12.6 g/dL) across N = 426 participants. (TIF) [file pone.0302883.s002.tif]
