# Supplementary material for: Prediction of anemia in real-time using a smartphone camera processing conjunctival images
Source: PLoS One. 2024 May 13;19(5):e0302883. doi: 10.1371/journal.pone.0302883 (PMC11090304; doi:10.1371/journal.pone.0302883)
Supplement: S3 Fig — The high hue ratio (HHR) primary parameter extracted from the images was the most predictive of HBI. HHR values were correlated with HBc range of 2 to 14 g/dL The mean HBl intercept was 5.8 (4.9, 6.7) (95% CI; p<0.001). The mean HHR was 7.6 (6.6, 8.5) (95% CI; p<0.001). Value of mean std error was 5.47 and R-squared was 0.372. (DOCX) [file pone.0302883.s003.docx]

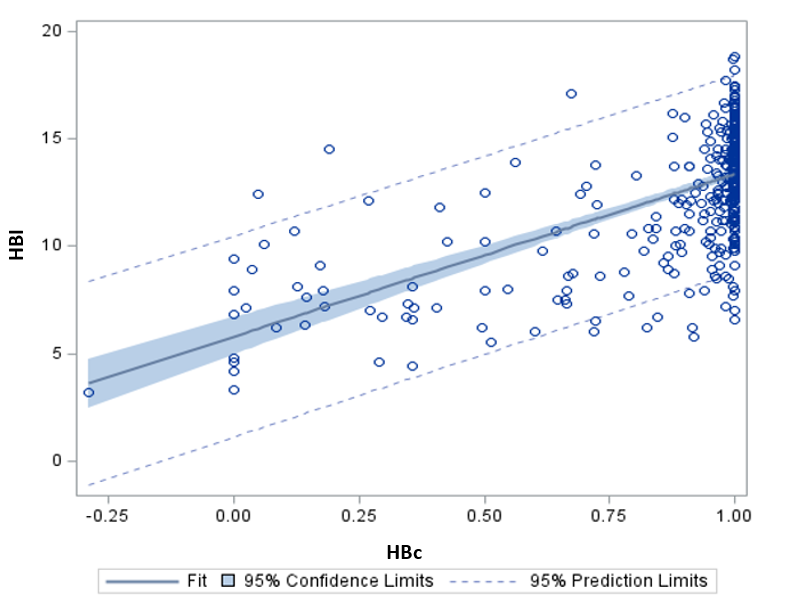


**HBl**

**Average HHR**

**The high hue ratio (HHR) primary parameter extracted from the images was the most predictive of HBI. HHR values were correlated with HBc range of 2 to 14 g/dL The mean HBl intercept was 5.8 (4.9, 6.7) (95% CI; p<0.001). The mean HHR was 7.6 (6.6, 8.5) (95% CI; p<0.001). Value of mean std error was 5.47 and R-squared was 0.372.**
