# Supplementary material for: Prediction of anemia in real-time using a smartphone camera processing conjunctival images
Source: PLoS One. 2024 May 13;19(5):e0302883. doi: 10.1371/journal.pone.0302883 (PMC11090304; doi:10.1371/journal.pone.0302883)
Supplement: S1 Table — (DOCX) [file pone.0302883.s004.docx]

| **Title** | **Author(s)** | **Modality**  **(Conjunctiva,**  **Skin, Nail bed)** | **Image Data** | **Programming Approach** | **Post-hoc Analysis** | **Real Time Analysis** | **Derivation Table**  **Population**  **Size** | **Validation Table**  **Population**  **Size** | **Accuracy Analysis** | **Sens/Spec** | **AUC** | **R-value** | **Reported**  **Accuracy or**  **LOA** | **PMID Number/doi number** |
| --- | --- | --- | --- | --- | --- | --- | --- | --- | --- | --- | --- | --- | --- | --- |
| **Smartphone app for non-invasive detection of anemia using only patient-sourced photos** | Mannino et al. | Fingernail Bed | Image Metadata | Multi-linear Regression | + | - | 100 | - | Bland-Altman Analysis | 92/76 | 0.88 | 0.82 | ±2.4 g/dL | PMID 30514831 |
| **Smartphone-based point-of-care anemia screening in rural Bihar in India** | Haggenmüller et al. | Fingernail Bed | Image Metadata | Multi-linear Regression | - | + | - | N = 272 adults & N= 179  Children | Bland-Altman Analysis | 51.2/41.6 | 0.65 | 0.225 | ±4.43 g/dL | PMID 36949164 |
| **HemaApp: noninvasive blood screening of hemoglobin using smartphone cameras** | Wang et al. | Volar Finger Tip | Infra-Red | Machine Learning | - | + | 20 | 32 | Bland-Altman Analysis | 85.7/76.5 | - | 0.69-0.82 | - | <https://doi.org/10.1145/2971648.2971653> |
| **Prediction of anemia and estimation of hemoglobin concentration using a smartphone camera** | Suner et al. | Conjunctiva | RAW image  files | Computer Vision  Algorithm 32 bits/pixel  (High Hue Ratio) | + | - | 142 | 344 | ROC Curves &  Bland-Altman Analysis | 72.8/72.5 | 0.8 | - | 72.60% | PMID 34260592 |
| **A Noninvasive Computerized Technique to Detect Anemia Using Images of Eye Conjunctiva** | Bauskar et al. | Conjunctiva | RGB image  files | Modified SVM Classifier | + | - | 99 | - | - | - | - | - | - | https://doi.org/10.1134/S1054661819030027 |
| **An intelligent non-invasive system for automated diagnosis of anemia exploiting a novel dataset** | Dimauro et al. | Conjunctiva | RGB image  files | Neural Network (RUSBoost) | + | - | 213 | - | - | 0.66/0.91 | - | - | 88% | PMID 36710064 |
| **Detection of anemia using conjunctiva images: A smartphone application approach** | Appiahene et al. | Conjunctiva | RGB image  files | Neural Network  (Convolutional Neural  Networks, Logistic regression, Classification, and Gaussian Blur) | + | + | 710 | 100 | - | 90/95 | - | - | 92.50% | https://doi.org/10.1016/j.medntd.2023.100237 |
| **Examining palpebral conjunctiva for anemia assessment with image processing methods** | Chen et al. | Conjuctiva | RGB image  files | Support Vector Machine or Neural Network | + | - | 100 | - | - | 0.62/0.90 | - | - | - | PMID 28110719 |
| **Feasibility of smartphone colorimetry of the face as an anaemia screening tool for infants and young children in Ghana** | Wemyss et al. | Conjunctiva, Lower Lip and Sclera | RGB image  files | Colorimetric Algorithm | + | - | 62 | - | Bland-Altman Analysis | 92.9/89.7 | 0.909 | - | ±3.36 g/dL | PMID 36867642 |
| **mHealth spectroscopy of blood hemoglobin with spectral superresolution** | Park et. al | Conjunctiva | RGB image  files | Spectral Super-resolution (SSR) | + | - | 138 | 15 | ROC Curves &  Bland-Altman Analysis | - | 0.98 | 0.96 | ±2.24 g/dL | PMID 33365364 |
| **Mobile Application for Anemia Detection through Ocular Conjunctiva Images** | Rivero-Palacio et al. | Conjunctiva | RGB image  files | Neural Network (YOLO) | + | - | 457 | - | - | 71.0/89.0 | - | - | - | doi:10.1109/ColCACI52978.2021.9469593 |
| **Neural network based non-invasive method to detect anemia from images of eye conjunctiva** | Jain et al. | Conjunctiva | RGB image  files | Neural Network | + | - | 99 | - | - | 99.21/95 | - | - | 97% | https://doi.org/10.1002/ima.22359 |
| **Non-Invasive Detection of Anaemia Using Digital Photographs of the Conjunctiva** | Collings et al. | Conjunctiva | RGB image  files | Colorimetric Algorithm (RGB) | + | - | 106 | - | ROC Curves | 0.74/0.71 | 0.86 | 0.63 | - | PMID 27070544 |
| **SmartHeLP: Smartphone-based Hemoglobin**  **Level Prediction Using an Artificial Neural Network** | Hasan et al. | Fingernail Bed | RGB image  files | Artificial Neural Network | + | - | 75 | - | - | 94/96 | - | - | - | PMID 30815094 |
