# Supplementary material for: Prediction of anemia in real-time using a smartphone camera processing conjunctival images
Source: PLoS One. 2024 May 13;19(5):e0302883. doi: 10.1371/journal.pone.0302883 (PMC11090304; doi:10.1371/journal.pone.0302883)
Supplement: S2 Table — (DOCX) [file pone.0302883.s005.docx]

|  | **mean (sd) or n (%)** |
| --- | --- |
| **Age, mean** | 52.6 (18.3) |
| **Age, group** |  |
| **1-21** | 10 (2.3%) |
| **22-64** | 289 (67.8%) |
| **≥65** | 127 (29.8%) |
| **Sex** |  |
| **Female** | 235 (55.2%) |
| **Male** | 191 (44.8%) |
| **Race** |  |
| **Asian** | 3 (0.7%) |
| **Black/African American** | 57 (13.4%) |
| **White/Caucasian** | 310 (72.8%) |
| **Multi-race** | 1 (0.2%) |
| **Other** | 51 (12%) |
| **Refused** | 4 (0.9%) |
| **Ethnicity** |  |
| **Hispanic or Latinx** | 54 (12.7%) |
| **Non-Hispanic or Latinx** | 364 (85.4%) |
| **Other** | 5 (1.2%) |
| **Refused** | 3 (0.7%) |
| **Skin Color** |  |
| **D** | 44 (10.3%) |
| **L** | 322 (75.6%) |
| **M** | 60 (14.1%) |
| **Jaundice** |  |
| **M** | 1 (0.2%) |
| **N** | 424 (99.6%) |
| **Y** | 1 (0.2%) |
| **SpO2, mean** | 97.5 (5.1) |
| **SpO2, group** |  |
| **﹥95** | 367 (86.2%) |
| **93-95** | 46 (10.8%) |
| **90-92** | 5 (1.2%) |
| **86-89** | 2 (0.5%) |
| **Unavailable** | 6 (1.4%) |

**Patient Demographics**
