# Supplementary material for: Prediction of anemia in real-time using a smartphone camera processing conjunctival images
Source: PLoS One. 2024 May 13;19(5):e0302883. doi: 10.1371/journal.pone.0302883 (PMC11090304; doi:10.1371/journal.pone.0302883)
Supplement: S3 Table — Accuracy, sensitivity, specificity, false positive rate, and false negative rate for anemia as defined by the American Society of Hematology (ASH) and for transfusion thresholds as defined by the Association for the Advancement of Blood & Biotherapies (AABB) are shown as predicted values [95% CI]. (DOCX) [file pone.0302883.s006.docx]

| Predicted Outcome | HBc predicting HBI | Estimate [95% CI] |
| --- | --- | --- |
| Anemia | Accuracy | 75.4 [71.3, 79.4] |
| <12 g/dL Women | Sensitivity | 54.3 [46.9, 61.8] |
| <13.5 g/dL Men | Specificity | 89.7 [86.0, 93.5] |
|  | False Positive Rate | 10.3 [6.5, 14.0] |
|  | False Negative Rate | 45.7 [38.2, 53.1] |
| Transfusion Low | Accuracy | 92.7 [90.3, 95.2] |
| <7 g/dL | Sensitivity | 69.6 [50.8, 88.4] |
|  | Specificity | 94.0 [91.7, 96.4] |
|  | False Positive Rate | 6.0 [3.6, 8.3] |
|  | False Negative Rate | 30.4 [11.6, 49.2] |
| Transfusion High | Accuracy | 91.1 [88.4, 93.8] |
| <9 g/dL | Sensitivity | 58.3 [45.9, 70.8] |
|  | Specificity | 96.5 [94.6, 98.3] |
|  | False Positive Rate | 3.6 [1.7, 5.4] |
|  | False Negative Rate | 41.7 [29.2, 54.1] |

**Hemoglobin values are in g/dL.**

**Clinical usefulness of conjunctiva estimated Hb (HBc). Accuracy, sensitivity, specificity, false positive rate, and false negative rate for anemia as defined by the American Society of Hematology (ASH) and for transfusion thresholds as defined by the Association for the Advancement of Blood & Biotherapies (AABB) are shown as predicted values [95% CI].**
