# Supplementary material for: Prediction of anemia in real-time using a smartphone camera processing conjunctival images
Source: PLoS One. 2024 May 13;19(5):e0302883. doi: 10.1371/journal.pone.0302883 (PMC11090304; doi:10.1371/journal.pone.0302883)
Supplement: S4 Table — Intraclass correlation calculation using a mixed effect regression analysis predicting laboratory-determined Hb (HBl) measurement using the eMoglobin data. (DOCX) [file pone.0302883.s007.docx]

| **RA** | **n (%)** |
| --- | --- |
| AC | 11 (2.6%) |
| AC/MJ | 2 (0.5%) |
| AF | 22 (5.2%) |
| AF/MJ | 4 (0.9%) |
| AM | 28 (6.6%) |
| AM/MS | 1 (0.2%) |
| BS | 84 (19.7%) |
| BS/JD | 2 (0.5%) |
| CH | 35 (8.2%) |
| FL | 21 (4.9%) |
| FL/ST | 1 (0.2%) |
| HG/ST | 1 (0.2%) |
| IF | 11 (2.6%) |
| JD | 6 (1.4%) |
| JK | 15 (3.5%) |
| JK/AM | 4 (0.9%) |
| LZ | 2 (0.5%) |
| MJ | 51 (12%) |
| MJ/AC | 2 (0.5%) |
| MS | 4 (0.9%) |
| SK | 27 (6.3%) |
| SK/FL | 2 (0.5%) |
| ST | 30 (7%) |
| ST/AF | 1 (0.2%) |
| VS | 56 (13.2%) |
| VS/AC | 1 (0.2%) |

**2-level re-categorization of RAs by volume**

| **Volume** | **n (%)** |
| --- | --- |
| < 20 | 72 (16.9%) |
| 20+ | 354 (83.1%) |

RA’s < 20: JK; MS; LZ; IF; AC; HG; AC/MJ; AF/MJ; AM/MS; BS/JD; FL/ST; HG/ST; JD; JK/AM; MJ/AC; SK/FL; ST/AF; VS/AC; VS/MJ

RA 20+: AF; AM; CH; FL; SK; ST; BS; VS; MJ

**Random effect for 2-cat (<20; 20+)**

| **Parameter** | **Estimate (95% CI)** | **p-value** |
| --- | --- | --- |
| **Intercept** | 4.26 (95% CI: -3.08, 11.59) | 0.08 |
| **eMoglobin Data** | 0.68 (95% CI: 0.61, 0.76) | < .001 |

Covariance: 0.1764 / (0.1764 + 4.9782) = 0.342 * 100 = 3.4%

**In this model using 2-level category for RA’s based on volume (< 20 patients, 20 or more), we found that 3.4% of the variance in eMoglobin data with the HBl data is explained by the RA performance.**

**Intraclass Correlation Calculation for RAs**

**Intraclass correlation calculation using a mixed effect regression analysis predicting laboratory-derived hemoglobin (HBl) measurement using the eMoglobin data.**
